# A Danish population-based cohort study of newly diagnosed asthmatic children's care pathway – adherence to guidelines

**DOI:** 10.1186/1472-6963-8-130

**Published:** 2008-06-12

**Authors:** Grete Moth, Peter O Schiotz, Peter Vedsted

**Affiliations:** 1 Danish Paediatric Asthma Centre, Aarhus University Hospital, Skejby, Denmark; 2 The Research Unit for General Practice, University of Aarhus, Denmark

## Abstract

**Background:**

Asthma is the most common chronic disease in childhood. Large variations exist concerning the number of children being treated by general practitioners and by specialists. Consequently, health related costs due to this disease vary as care by specialists is more expensive compared with care by general practitioners. Little is known of the consequences of these variations concerning the quality of care. The aim of the study was to analyse associations between care providers and adherence to guidelines concerning frequency of contacts with the health service due to asthma.

**Methods:**

A cohort study was performed of 36,940 incident asthmatic children's (aged 6–14) contacts with the health service using the unique personal registration number to link data from five national registries. The prevalence ratios were calculated for associations between provider (general practitioner, primary care specialist, hospital specialist or both GP and specialist) and adherence with guidelines concerning three indicators of quality of care pathway: 1) diagnostic examination of lung function at start of medical treatment 2) follow-up the first six months and 3) follow-up the next six months. The associations were adjusted for sex, age, socioeconomic status, county, and severity of disease.

**Results:**

Most children (70.3%) had only been seen by their GP. About 80% of the children were treated with inhaled steroids, 70% were treated with inhaled steroids as well as inhaled beta2agonists and 13% were treated with inhaled beta2agonists only. A total of 12,650 children (34.2%) had no registered asthma-related contacts with the health service except when redeeming prescriptions. Care was in accordance with guidelines in all three indicators of quality in 7% of the cases (GPs only: 3%, primary care specialists only: 16%, hospital specialists: 28%, and both GP and specialists: 13%). Primary care specialists had a 5.01, hospital specialists a 8.81 and both GP and specialists a 4.32 times higher propensity to provide a clinical pathway according to guidelines compared to GPs alone.

**Conclusion:**

The majority of the children were seen in general practice. Hospital specialists provided care in accordance with guidelines nine times more often compared with GPs, but still only one quarter of these children had pathways in accordance with guidelines. It is relevant to study further if these lacks of adherence to guidelines have implications for the asthmatic children or if guidelines are too demanding concerning frequency of follow-up or if asthmatic children should be stratified to different care pathways.

## Background

Asthma is the most common chronic disease in childhood and the associated public health expenditure is high [1-5]. Ideally, care for asthmatic children is carried out according to international and national guidelines, as care complying with guidelines is related to improved patient outcome [6,7]. According to guidelines care of children with mild asthma can be managed by general practitioners (GPs) [8-10]. This is in line with the explicit intention of the overall health plans of the Danish counties that health care is to be organized with mild cases managed by GPs and more severe or problematic cases are referred to specialists. This is to ensure the most efficient use of the available health resources. In practice, paediatric asthma care is managed within a framework defined by traditions, guidelines, and available resources depending on political and administrative decisions in the counties concerning organization of health care and the division of care between general practice, specialists in primary health care, and hospitals.

However, despite intentions to organize care in the most efficient way, there are considerable differences concerning the number of asthmatic children being treated by GPs and by specialists internationally [11,12] as well as in Denmark. This is shown in previous studies by our group [13,14]. These variations indicate systematic differences in the organization of health care for asthmatic children. This is interesting in the perspectives of health services research and quality of care. Therefore, it is relevant to study these differences in care pathways of asthmatic children in order to measure the impact of care providers on quality of care.

In Denmark it is possible to study care pathways in a nationwide scale due to existence of national registries and the unique personal registration number (PRN), assigned to all citizens [15]. The PRN permits data from the national registries containing data on all Danish citizens at an individual level to be linked [16-18].

### Aim

The aim of this study was to map out incident asthmatic children's care pathways six months prior to and the first year following start of medical treatment to measure associations between 1) characteristics of the children and their care pathways and 2) type of care provider and adherence to guidelines.

## Methods

A historical population-based cohort study was conducted of asthmatic children's care pathways for 18 months using registry data linked from five national registries.

### Study population

Children born between 1988 and 1996 using anti-asthmatic medication in the period from 1996 throughout 2004 formed a cohort of 6–14-year-old asthmatic children. We used a validated method based on redeemed prescriptions described elsewhere [19] to include asthmatic children. A short presentation of the method is given in Appendix 1. Focusing on incident asthmatic children we restricted inclusion to children that redeemed anti-asthmatic medication for the first time between 1999 and 2004 or after a medication free period of at least two years. Children that moved between counties during the observation period were excluded.

### Study period

The asthmatic children were followed from six months before until 12 months after starting their anti-asthmatic treatment in the period from 1999 throughout 2004.

### Description and categorization of data

Data on anti-asthmatic medication were identified by the ATC codes described in Appendix 2 and were obtained from the Register of Medicinal Product Statistics [17]. Data obtained included: the PRN number, ATC code, number of packages, and date of dispensing. Use of packages was summed up for each month for the following five types of medication: inhaled steroid, inhaled short-acting beta2agonist, inhaled long-acting beta2agonist, fixed combinations of inhaled steroid and long-acting beta2agonist, and leukotrien receptor antagonist. Severity of disease categories were based on number of packages in the study period: mild: 1–4, moderate: 5–8, and severe: > 8.

Data from the National Danish Patient Registry [16] on visits to outpatient clinics and hospital admissions due to one of the asthma diagnoses shown in Appendix 3 were summed up for each month.

In Denmark every child is registered with a GP and this GP is gatekeeper to the rest of the health care system. Contacts with GPs and primary care specialists were also summed up for each month. As these are not registered with a diagnosis in the National Health Insurance Service Registry [18] but by the performed examination, we decided to include contacts that were registered by a peakflow test, a spirometry, or a reversibility test as a proxy for a visit due to asthma.

For each child the visits to GPs and specialists in primary care and the contacts to hospitals were used to define the provider that had managed the care of the child during the whole 18 months study period (six months prior to and 12 months after medication start). The study population was divided into four groups depending on provider of asthma care during the whole study period:

1) Contacts only with GP.

2) Contacts only with specialist in the primary health care sector.

3) Contacts only with specialist at out-patient clinic/hospital.

4) Contacts with GP as well as specialists.

Thus, if a child had a contact to the GP at start of medication and no further contacts the GP was defined as the care provider for the whole period. If medication was initiated by a specialist and the child had performed a lung function test at the GPs at some time during the observation period the child was placed in provider group 4.

Data on parent's marital status (single or cohabiting/married), education level, and annual income were acquired for the year of inclusion from Statistics Denmark [20]. In case of divorce the child was presumed to live with the mother, as is the case for more than 90% of children of divorced parents in Denmark [21]. Data on education of the parents were categorized into three groups: short: primary and high school (< 14 years), intermediate: short or intermediate higher education (14–18 years), and long: higher education (> 18 years). Likewise, data on income were categorized in four groups 1: < 150,000 (approximately equivalent to unemployment benefit (20,000 Euro)). 2: 150,000–250,000. 3: 250,000–350,000. 4: > 350,000 (DKK) (1 Euro = 7.5 DKK).

### Guidelines

Recommendations concerning asthmatic children's care pathway were extracted from the GINA-guidelines and the British and Canadian national guidelines for paediatric asthma [8-10]. We focused on:

1) Diagnostic examination of lung function during the 6-month period before or at start of medical treatment.

2) Follow-up at least once during the first six months following start of medical treatment.

3) Follow-up at least once during the next six months.

An overall indicator of quality of care was applied based on whether all the indicators 1–3 were achieved.

### Analysis

Associations between characteristics of the children (gender, age, socioeconomic factors, and disease severity) and adherence to guidelines were calculated by use of a generalized linear model (GLM). Further, we calculated the association between care provider and the adherence to guidelines adjusted for the children's gender, age, county, socioeconomic status, and severity of disease. Associations were calculated as the prevalence difference (PD) and the prevalence ratio (PR) between care providers. The PR is the ratio between the prevalences where the odds ratio is the ratio between odds. We preferred the PR to the odds ratio because the prevalences were high (> 20%) and the odds ratios therefore would tend to overestimate the PR [22]. Analyses were performed by use of STATA 9.

### Ethics

All data were stored at Statistics Denmark. We obtained access to an encrypted version of the data after approval by The Danish Data Protection Agency, the Danish National Board of Health, and Statistics Denmark (Project no. 702063).

## Results

During the period 1999–2004 anti-asthmatic drugs were dispensed to 46,043 6–14 year-old asthmatic children. Of these, 37,283 (81.0%) were incident asthmatics. Due to change of address between counties 343 children were excluded leaving 36,940 children for analyses.

Characteristics of the children in the cohort are presented in Table 1. Most of the children (70.3%) had only been treated by their GP including 12,650 children (34.2%) who had no registered asthma related contacts with the health service except for prescriptions for anti-asthmatic medication. About 80% were treated with inhaled steroids and 70% had been prescribed inhaled beta2agonists as well. Approx. 13% were treated with inhaled beta2agonists only. Half of the children were categorised as mild cases of asthma and 36% and 15% as moderate and severe asthma, respectively.

**Table 1:** Characteristics of children in the cohort (n = 36,940)

			**No. of children (%)**
**Demographic**	Gender	Boys	22,480 (60.9)
		Girls	14,460 (39.1)
	Age at inclusion (mean: 9.2 years)	6–8 years	18,025 (48.8)
		9–11 years	14,174 (38.4)
		12–13 years	4,741 (12.8)

**Socioeconomic status**	Family characteristic in the year of inclusion	Two-parent family	30,120 (81.6)
		Single-parent family	6,662 (18.0)
		No information	158 (0.4)
	Parent's education level	1. Primary and high school	21,998 (59.6)
		2. Short or intermediate higher education	10,194 (27.6)
		3. Long higher education	4,590 (12.4)
		No information	158 (0.4)
	One parent's income in the year of inclusion (DKK)	1. < 150,000	5,659 (15.3)
		2. 150,000–250,000	13,733 (37.2)
		3. 250,000–350,000	12,000 (32,5)
		4. > 350,000	5,389 (14.7)
		No information	159 (0.3)

**Geograhpy**	Distribution of children in the counties	Mean (min – max)	2,639 (196 – 6,675)
		Overall share of asthmatic children (min-max)	4.1% (3.8 – 7.4)

**GPs or specialists as healthcare providers in the study period**	Contact only with GPs with examination of lung function		13,316 (36.1)
	Contact only with GPs without examination of lung function		12,650 (34.2)
	Contact only with primary care specialists		1,286 (3.5)
	Contact only with specialists at outpatient clinics		2,594 (7.0)
	Contact with both GPs and specialists		7,094 (19.2)

**Medical treatment in 12 months**	Inhaled steroids		29,912 (81.0)
	Inhaled short-acting beta2 agonists		29,741 (80.5)
	Long-acting beta2 agonists		5,225 (14.2)
	Inhaled steroids and long-acting beta2 agonists in a fixed combination		2,781 (7.5)
	Leukotrien receptor antagonists		1,437 (3.9)
	Inhaled steroids only		3,547 (9.6)
	Inhaled beta2 agonists only		4,857 (13.2)
	Inhaled steroids and beta2 agonists (incl. fixed combinations of steroid and long-acting beta2agonists)		25,755 (69.7)

**Severity of disease**	No. of packages of anti-asthmatic drugs:		
	Mild: ≤ 4		18,046 (48.8)
	Moderate: 5–8		13,290 (36.0)
	Severe: > 8		5,604 (15.2)

In Table 2, 3, 4 the association between child characteristics of the children and adherence to guideline is shown. The more severe the asthma was, the more often the lung function test was done. Children aged 9–11 and 12–14 years had a higher propensity to receive a lung function test in the diagnostic period and during the first six months of follow up compared with 6–8 year-old children. Concerning socioeconomic factors children from low income families had a small but statistically significantly lower propensity of receiving a diagnostic lung function test and follow-up during the first six months. Children from two-parent families were more often monitored with follow-up visits compared with single-parent children.

**Table 2:** Association between characteristics of children and quality of care measured by lung function tests at start of medical treatment (n = 36,781)

**Examination of lung function at start of medical treatment**							
			P (No.)	PD (95% CI)	PR (95% CI)	Adj. PR (95% CI)	P-values

**Gender**	Girl		0.42 (6,107)	ref	1	1	
	Boy		0.43 (9,656)	0.01 (-0.03–0.02)	1.02 (0.99–1.04)	1.02 (0.99–1.04)	0.170

**Age**	6–8		0.39 (7,104)	ref	1	1	
	9–11		0.45 (6,369)	0.06 (0.04–0.07)	1.14 (1.11–1.17)	1.15 (1.12–1.18)	< 0.001
	12–14		0.48 (2,290)	0.09 (0.07–0.11)	1.23 (1.18–1.27)	1.26 (1.22–1.3)	< 0.001

**SES**	Single		0.42 (2,797)	ref	1		
	Cohabiting		0.43 (12,914)	0.01 (-0.0–0.02)	1.02 (0.99–1.05)	1.00 (0.96–1.03)	0.748
	Education	1	0.43 (9,347)	ref	1	1	
		2	0.43 (4,425)	0.01 (0.0–0.02)	1.02 (0.99–1.05)	1.00 (0.97–1.03)	0.923
		3	0.42 (1,939)	0.0 (-0.02–0.01)	0.99 (0.96–1.03)	1.0 (0.97–1.03)	0.838
	Income	1	0.41 (2,297)	ref	1		
		2	0.43 (5,840)	0.02 (0.0–0.04)	1.05 (1.01–1.09)	1.04 (1.0–1.08)	0.050
		3	0.44 (5,268)	0.03 (0.02–0.05)	1.08 (1.04–1.12)	1.07 (1.03–1.12)	< 0.001
		4	0.43 (2,306)	0.02 (0.0–0.04)	1.05 (1.01–1.10)	1.05 (1.01–1.1)	0.029

**Severity of disease**	Mild		0.40 (7,162)	ref	1	1	
	Moderate		0.45 (5,927)	0.05 (0.04–0.06)	1.12 (1.1–1.15)	1.13 (1.1–1.16)	< 0.001
	Severe		0.48 (2,674)	0.08 (0.07–0.1)	1.20 (1.16–1.24)	1.22 (1.18–1.26)	< 0.001

**Table 3:** Association between characteristics of children and quality of care measured by lung function tests during follow-up in the first six months after start of medication (n = 36,781)

**Follow up 1–6 months after start of medical treatment**							
			P (No.)	PD (95% CI)	PR (95% CI)	Adj. PR (95% CI)	P-values

**Gender**	Girl		0.42 (6,008)	ref	1	1	
	Boy		0.41 (9,298)	0.00 (-0.01–0,01)	1.00 (0.97–1.02)	0.99 (0.97–1.01)	0.353

**Age**	6–8		0.39 (6,994)	ref	1	1	
	9–11		0.44 (6,170)	0.05 (0.04–0.06)	1.12 (1.09–1.15)	1.14 (1.11–1.17)	< 0.001
	12–14		0.45 (2,142)	0.06 (0.05–0.08)	1.17 (1.12–1.21)	1.23 (1.19–1.27)	< 0.001

**SES**	Single		0.40 (2,655)	ref	1	1	
	Cohabiting		0.42 (12,612)	0.02 (0.01–0.03)	1.05 (1.02–1.09)	1.03 (0.99–1.06)	0.004
	Education	1	0.42 (9,130)	ref	1	1	
		2	0.42 (4,271)	0.00 (-0.01–0.02)	1.01 (0.98–1.04)	0.98 (0.95–1.01)	0.148
		3	0.41 (1,866)	0.0 (-0.02–0.01)	0.98 (0.94–1.02)	0.97 (0.93–1.01)	0.114
	Income	1	0.39 (2,202)	ref	1	1	
		2	0.41 (5,686)	0.03 (0.01–0.04)	1.06 (1.02–1.11)	1.05 (1.01–1.09)	0.019
		3	0.43 (5,126)	0.04 (0.02–0.05)	1.10 (1.06–1.14)	1.08 (1.04–1.12)	< 0.001
		4	0.42 (2,252)	0.03 (0.01–0.05)	1.07 (1.03–1.12)	1.07 (1.02–1.12)	0.008

**Severity of disease**	Mild		0.35 (6,223)	ref	1	1	
	Moderate		0.47 (6,208)	0.12 (0.11–0.13)	1.36 (1.32–1.39)	1.37 (1.33–1.4)	< 0.001
	Severe		0.51 (2,875)	0.17 (0.15–0.18)	1.49 (1.44–1.53)	1.51 (1.46–1.56)	< 0.001

**Table 4:** Association between characteristics of children and quality of care measured by lung function tests during follow-up in the months 7–12 after start of medication (n = 36,781)

**Follow up 7–12 months after start of medical treatment**							
			P (No.)	PD (95% CI)	PR (95% CI)	Adj. PR (95% CI)	P-values

**Gender**	Girl		0.23 (3,317)	ref	1	1	
	Boys		0.25 (5,599)	0.02 (-0.0–0.03)	1.09 (1.05–1.13)	1.05 (1.01–1.09)	0.008

**Age**	6–8		0.24 (4,380)	ref	1	1	
	9–11		0.25 (3,520)	0.01 (0.0–0.05)	1.02 (0.98–1.06)	1.07 (1.03–1.11)	0.001
	12–14		0.21 (1,016)	-0.03 (-0.04- -0.02)	0.88 (0.83–0.94)	1.01 (0.95–1.07)	0.767

**SES**	Single		0.23 (1,515)	ref	1	1	
	Cohabiting		0.25 (7,389)	0.02 (0.01–0.03)	1.08 (1.03–1.13)	1.06 (1.01–1.11)	0.020
	Education	1	0.24 (5,213)	ref	1	1	
		2	0.25 (2,542)	0.01 (0.0–0.02)	1.05 (1.01–1.1)	1.01 (0.97–1.06)	0.627
		3	0.25 (1,149)	0.01 (0.0–0.03)	1.06 (1.0–1.12)	1.05 (0.99–1.11)	0.102
	Income	1	0.24 (1,378)	ref	1	1	
		2	0.24 (3,319)	0.00 (-0.02–0.01)	0.99 (0.94–1.05)	0.97 (0.91–1.02)	0.189
		3	0.25 (2,942)	0.00 (-0.01–0.02)	1.01 (0.95–1.06)	0.97 (0.92–1.03)	0.288
		4	0.24 (1,265)	-0.01 (-0.03–0.01)	0.96 (0.90–1.03)	0.92 (0.86–0.99)	0.018

**Severity of disease**	Mild		0.14 (2,577)	ref	1	1	
	Moderate		0.31 (4,090)	0.17 (0.16–0.17)	2.16 (2.06–2.25)	2.15 (2.06–2.24)	< 0.001
	Severe		0.40 (2,249)	0.26 (0.25–0.27)	2.81 (2.68–2.95)	2.79 (2.66–2.93)	< 0.001

Table 5 shows the association between quality and provider of care with GPs as the reference group. The prevalence of overall quality of care was in accordance with guidelines for 3%, 16%, 28%, and 13% of the children cared for by GPs only, by primary care specialist only, by specialists at hospitals only or by both GPs and specialists, respectively. The PR of managing a care pathway according to guidelines by primary care specialists compared to GPs was 5.01 and by hospital specialists compared to GPs PR was 8.81 and by both specialists and GP PR was 4.32. When excluding the 12,650 children without lung-specific contacts with the health service in order to compare GPs that actually performed examinations and monitored the children with specialist care, the associations for having a care pathway according to guidelines were reduced to 2.62 (2.62–3.04) for primary care specialists, and to 4.59 (4.16–5.06) for hospital specialists compared with GPs, and for both specialists and GP compared with GPs only PR was 2.26 (2.05–2.48). Accordance with guidelines on all three indicators of care pathway was the case for 9.2% of the children.

**Table 5:** Association between quality of care (adherence to guidelines measured by three single indicators and overall) and provider of care (n = 36,781)

		**Indicators of quality of treatment**			
		**Examination of lung function prior to or at start of med. treatment**	**Follow-up 1–6 months after start of med. treatment**	**Follow-up 7–12 months after start of med. treatment**	**Overall quality of care **(all three indicators)
**GPs **(Ref) n = 25,843	**P (No.)**	0.34 (8,926)	0.30 (7,693)	0.11 (2,786)	0.03 (692)

**Primary care specialists **n = 1,282	**P (No.)**	1.00 (1,286)	0.52 (666)	0.32 (406)	0.16 (201)
	**PD (95% CI)**	0.66 (0.65–0.66)	0.22 (0.19–0.25)	0.21 (0.18–0.23)	0.13 (0.11–0.15)
	**PR (95% CI)**	2.91 (2.86–2.96)	1.75 (1.65–1.85)	2.94 (2.70–3.21)	5.87 (5.07–6.79)
	**Adj PR (95% CI)**	2.88° (2.82–2.93)	1.68 (1.59–1.78)	2.59 (2.38–2.83)	5.01 (4.32–5.80)
	**P-values**	< 0.001	< 0.001	< 0.001	< 0.001

**Specialists at hospital **n = 2,580	**P (No.)**	1.00 (2.594)	0.57 (1,488)	0.54 (1,406)	0.28 (716)
	**PD (95% CI)**	0.66 (0.65–0.66)	0.28 (0.26–0.30)	0.44 (0.42–0.45)	0.25 (0.23–0.27)
	**PR (95% CI)**	2.92 (2.86–2.96)	1.94 (1.86–2.01)	5.05 (4.81–5.31)	10.4 (9.41–11.4)
	**Adj PR (95% CI)**	2.98 (2.93–3.04)	1.91 (1.84–1.99)	4.48 (4.26–4.72)	8.81 (7.98–9.73)
	**P-values**	< 0.001	< 0.001	< 0.001	< 0.001

**GPs and specialists **n = 7,070	**P (No.)**	0.42 (2,957)	0.77 (5,459)	0.61 (4,318)	0.13 (949)
	**PD (95% CI)**	0.08 (0.06–0.09)	0.47 (0.46–0.49)	0.50 (0.49–0.51)	0.11 (0.10–0.12)
	**PR (95% CI)**	1.21 (1.17–1.25)	2.60 (2.54–2.66)	5.67 (5.45–5.90)	5.02 (4.57–5.52)
	**Adj PR (95% CI)**	1.22 (1.18–1.26)	2.52 (2.46–2.58)	5.04 (4.84–5.26)	4.32 (3.92–4.76)
	**P-values**	< 0.001	< 0.001	< 0.001	< 0.001

° Poisson regression

Adjusted for age, gender, socioeconomic status, county, and severity of disease

P: Prevalence, PD: Prevalence difference, PR: Prevalence ratio

## Discussion

### Main findings

Evaluated on the basis of three quality indicators concerning care pathway 3% of the asthmatic children had been followed according to guidelines by GPs compared with 16% and 28% of children followed by primary care specialists and hospital specialists, respectively. Therefore, care by primary care specialists, by hospital specialists and by GPs and specialists in combination was 5.01, 8.81, and 4.32 times more associated with care in accordance with guidelines compared with care by GPs only. Older children received more diagnostic lung function tests and initial follow up than younger children. Diagnostic lung function tests and initial follow up according to guidelines was more often the case for older children and for children from families with a higher income. Children from two-parent families were more often monitored with follow-up visits compared with single-parent children. Children with moderate or severe asthma were more likely to have a care pathway in accordance with guidelines than children with mild asthma.

### Strengths, limits, and bias

The method to identify the population of asthmatic children (Appendix 1) has been shown to have a sensitivity of 64% which might introduce selection bias in direction of including those with most severe asthma. However, with a specificity of 92% [19] the excluded children can be assumed to have mild or no asthma at all. Thus, including these children may have implied more serious selection bias and problems with generalisability.

Data on paediatric asthma diagnoses in the Danish National Patient Registry have been examined recently and found valid [23]. Data on contacts with GPs and specialists are based on an electronic registration of remunerated services carefully registered by the provider. Data on use of medication are based on the automatically generated list from all pharmacies of dispensed medicine to patients. The aggregated monthly health service use ensured a reasonable measure to report without losing too much information.

According to the guidelines the diagnosis of asthma is primarily based on the medical history but lung function tests are recommended to confirm the diagnosis and to monitor in the course of asthma. In the study we primarily wanted to map out the asthma related care pathways of the children using registry data and this was simple for the contacts to hospitals as these were registered by a diagnosis. However, lack of diagnoses linked to children's visits in the primary health care sector forced us to use data on examinations of lung function only

This means that we have missed follow-ups that were carried out without examinations of lung function. Consequently, we have underestimated the number of visits to GPs. It can be discussed whether a lung function test is needed to diagnose and monitor asthma, or if a thorough-medical history might be as fruitful. However, as approximately 90 % of Danish GPs have spirometry equipment and peakflow meters are available free to some GPs and at a low cost to others it is likely that a knowledgeable doctor would want an objective measure of a lung function. Thus, although we might have missed some follow-up visits we believe that lung function measurements are a suitable proxy for an asthma follow-up in compliance with the guidelines.

Our result that older children receive more tests of lung function than the 6–8-year-olds may be explained by the doctors not expecting the young children to be able to manage the technique. However, as peakflow test and spirometry is recommended from the age of 5–6 according to guidelines, the indicator was applied for this age-group as well.

We did not use Defined Daily Dose (DDD) to measure medication use as DDDs is the documented standard dose for adults. Furthermore, summing up DDDs would be meaningless, as the five categories of anti-asthmatic medication in the present study are made up by different types of medicine with different DDDs. Instead, we calculated the sum of packages to estimate severity of asthma.

Clearly, the three indicators do not represent a proper standard in cases of severe and persistent asthma. These cases are often referred to specialist care and by definition monitored more closely. This implies a problem of case mix in the present study, as GPs have a larger share of uncomplicated cases and thereby more easily risk exceeding six months between checks. Clinical data were not available to adjust for case mix due to severity of disease which even in clinical studies is complicated [24,25]. However, we used the number of packages of anti-asthmatic medication dispensed to the children as a proxy of severity of asthma in order to adjust for it. The chosen variable of severity is obviously dependent upon the children's adherence to the treatment and may as such to some degree be an expression of this. In case of this a child reimbursing too few drugs will be misclassified. However, a child not adhering to the treatment of inhaled steroid, which may often be the case, can be expected to need more drug of the reliever type and hence it will be classified as a more severe case anyway. The categories of severity showed good consistency with the clinical pathways found indicating that they to some degree serve the purpose. Such misclassification would tend to diminish the association towards no association

Though the diagnosis of asthma is primarily based on the medical history and the effect of lung function tests can be discussed these objective measures provide complementary information valuable in diagnosing and monitoring different aspects of asthma control according to the guidelines. As the aim of the study was to relate the care pathways of the children to the guidelines these recommendations of lung function tests were included.

It is worth noticing that concerning frequency of visits the guidelines state that the condition and treatment of asthmatic children are to be reviewed every six months as a minimum. Due to lack of research studies into this area this recommendation is not based on evidence but is presumably an expression of the general consensus on this subject derived from clinical experiences. However, despite the lack of evidence not mentioning the importance of follow-up would be a mistake considering the chronic and fluctuating nature of the disease. Schatz et al. in their study of frequency of follow-up visits concluded that adults with moderate persistent asthma did not need follow-up visits more often than every six months [26] Unlike this the present study can be expected to include also the mild persistent asthma cases due to the identification method [19]. Consequently, it may be a matter of discussion if lack of accordance with guidelines concerning the mild cases implies poor quality. In this respect it is worth considering that guidelines are developed by specialists with thorough knowledge of and experience with asthma in the most severe cases. In this study, although the very mild cases were excluded beforehand, specialists had been in contact with only 30% of the children. Consequently, the asthmatic children consulting specialists is a small and selected group with more severe asthma. So, the question is if guidelines on frequency of follow-up are relevant for all asthmatic children. It is especially worth considering if the required frequency of follow-up is reasonable or if asthmatics with mild intermittent and mild persistent asthma can manage with less frequent visits. Therefore, it may be more cost-effective to stratify children with asthma to different pathways according to their needs.

## Conclusion

In this study, adherence to guidelines concerning diagnosis and frequency of follow-up for Danish asthmatic schoolchildren were achieved in 7% of the cases. Care by hospital specialists alone was nine times more in accordance with guidelines compared with care by GPs. However, even in the hands of hospital specialists, all three guideline standards for care were met in only one fourth of the cases. The results of this study have lead to another study from our group on whether lack of correspondence with guidelines concerning care pathways as defined in this study implies an actual risk for the asthmatic children of being admitted to hospital due to asthma.

## Abbreviations

GP: General Practitioner; GINA: Global Initiative for Asthma; PNR: Personal Registration number; DNPR: the Danish National Patient Registry; ATC: Anatomical Therapeutic Chemical classification; GLM: Generalized Linear Model; P: Prevalence; PD: Prevalence difference; PR: Prevalence ratio.

## Competing interests

The authors declare that they have no competing interests.

## Authors' contributions

GM had primary responsibility for protocol development, for collecting data, for data analysis, and for writing the manuscript. POA supervised the design and execution of the study, and contributed to the writing of the manuscript. PV contributed by participating in the development of the design of the study, in the data analysis, and contributed to the writing of the manuscript. All authors read and approved the final manuscript.

## Appendix 1

### Description of the method to identify a population of asthmatic children

A register-based study of 125,907 6–14 year-old Danish children identified 9,695 children who had redeemed at least one anti-asthmatic drug prescription in 2002. The asthma diagnosis in these children was validated by discharge information from the Danish National Patient Registry and by general practitioners based on a questionnaire in which they were asked whether the children in question listed with their practice according to his/her medical knowledge had the diagnosis of asthma. Models based on combinations of different types of drugs were tested to find the best model including as many children with a validated diagnosis as possible and excluding as many false positives as possible. Different time windows were tested both concerning detecting the children and concerning observation period of redeeming prescriptions.

The highest specificity of 0.86 (CI: 0.84–0.87) together with a sensitivity of 0.63 (CI: 0.62–0.65) was seen in the model that included children that during a 12-month period had redeemed a prescription of any anti-asthmatic drug except beta2-agonist as liquid and except inhaled beta2 agonist or inhaled steroid only once. The positive predictive value of the method was 0.92, meaning that 92% of the children identified by this method actually had the diagnosis. Adding six months observation time did not improve the specificity significantly (0.87 (CI: 0.85–0.88)) but resulted in a statistically significantly lower sensitivity (0.59 (CI: 0.58–0.60)) [19].

## Appendix 2

### Asthma diagnoses (ICD10) of data from The National Patient Registry [27]

Asthma: J45. Predominantly allergic asthma: J45.0. Nonallergic asthma: J45.1. Mixed asthma: J45.8. Asthma, unspecified: J45.9. Status asthmaticus: J46.9

## Appendix 3

### Anti-asthmatic drugs listed by ATC-codes for data on use of medication [28]

Inhaled steroid: R03BA01, R03BA02 and R03BA05. Inhaled shortacting beta2 agonists: R03AC02, R03AC03 and R03AC04. Longacting beta2 agonists: R03AC13 and R03AC12. Inhaled steroid and longacting beta2 agonists in a fixed combination: R03AK06 and R03AK07. Leukotrien receptor antagonists: R03DC03

## Pre-publication history

The pre-publication history for this paper can be accessed here:



## References

[B1] Weiss KB, Sullivan SD (2001). The health economics of asthma and rhinitis. I. Assessing the economic impact. J Allergy Clin Immunol.

[B2] von Mutius E (2000). The burden of childhood asthma. Arch Dis Child.

[B3] Mossing R, Nielsen GD (2003). Cost-of-illness of asthma in Denmark in the year 2000 (in Danish). Ugeskr laeger.

[B4] (1998). Worldwide variation in prevalence of symptoms of asthma, allergic rhinoconjunctivitis, and atopic eczema: ISAAC. The International Study of Asthma and Allergies in Childhood (ISAAC) Steering Committee. Lancet.

[B5] Thomsen SF, Ulrik CS, Larsen K, Backer V (2004). Change in prevalence of asthma in Danish children and adolescents. Ann Allergy Asthma Immunol.

[B6] Grimshaw JM, Russell IT (1993). Effect of clinical guidelines on medical practice: a systematic review of rigorous evaluations. Lancet.

[B7] Glasgow NJ, Ponsonby AL, Yates R, Beilby J, Dugdale P (2003). Proactive asthma care in childhood: general practice based randomised controlled trial. BMJ.

[B8] (2007). Global Initiative for Asthma. http://www.ginasthma.com.

[B9] (2007). British Guideline on the Management of Asthma. http://www.sign.ac.uk/guidelines/fulltext/101/index.html.

[B10] (2007). Canadian Asthma Consensus Guidelines. http://www.cmaj.ca/cgi/reprint/161/11_suppl_1/s1.pdf.

[B11] Moore AT, Roland MO (1989). How much variation in referral rates among general practitioners is due to chance?. BMJ.

[B12] O'Donnell CA (2000). Variation in GP referral rates: what can we learn from the literature?. Fam Pract.

[B13] Moth G, Modlock J (2004). Differences among Danish counties in the use of specialist resources in the management of childhood asthma (In Danish). Ugeskr laeger.

[B14] Moth G, Schiøtz PO (2006). Hospital admission rates of Danish children with asthma in 2001: a register-based study (In Danish). Ugeskr laeger.

[B15] Pedersen CB, Gotzsche H, Moller JO, Mortensen PB (2006). The Danish Civil Registration System. A cohort of eight million persons. Dan Med Bull.

[B16] Andersen TF, Madsen M, Jørgensen J, Mellemkjær L, Olsen JH (1999). The Danish National Hospital Register. A valuable source of data for modern health sciences. Dan Med Bull.

[B17] Sørensen HT, Larsen BO (1994). A Population-Based Danish Data Resource with Possible High Validity in Pharmacoepidemiological Research. J Med Syst.

[B18] Olivarius NF, Hollnagel H, Krasnik A, Pedersen PA, Thorsen T (1997). The Danish National Health Service Register. Dan Med Bull.

[B19] Moth G, Vedsted P, Schiotz P (2007). Identification of asthmatic children using prescription data and diagnosis. Eur J Clin Pharmacol.

[B20] Thygesen L (1995). The register-based system of demographic and social statistics in Denmark - an overview. Stat J UN ECE.

[B21] (2007). Statistics Denmark.. http://www.statistikbanken.dk.

[B22] Kirkwood BR, Sterne JAC (2003). Essential Medical Statistics.

[B23] Moth G, Vedsted P, Schiotz P (2007). National registry diagnoses agree with medical records on hospitalized asthmatic children. Acta Paediatr.

[B24] Yawn BP, Brenneman SK, len-Ramey FC, Cabana MD, Markson LE (2006). Assessment of asthma severity and asthma control in children. Pediatrics.

[B25] Horner SD, Kieckhefer GM, Fouladi RT (2006). Measuring asthma severity: instrument refinement. J Asthma.

[B26] Schatz M, Rodriguez E, Falkoff R, Zeiger RS (2003). The relationship of frequency of follow-up visits to asthma outcomes in patients with moderate persistent asthma. J Asthma.

[B27] (2006). WHO The international statistical classification of diseases. http://www.who.int/classifications/icd/en/.

[B28] (2006). WHO Collaborating Centre for Drug Statistics Methodology. http://www.whocc.no.

